# Ethnoveterinary plants and practices used for ecto-parasite control in semi-arid smallholder farming areas of Zimbabwe

**DOI:** 10.1186/s13002-015-0006-6

**Published:** 2015-04-30

**Authors:** Emmanuel Tendai Nyahangare, Brighton Marimanzi Mvumi, Tonderai Mutibvu

**Affiliations:** Department of Animal Science, Faculty of Agriculture, University of Zimbabwe, P O Box MP167, Mt Pleasant, Harare Zimbabwe; Department of Soil Science and Agricultural Engineering, Faculty of Agriculture, University of Zimbabwe, P O Box MP 167 Mt Pleasant, Harare Zimbabwe

**Keywords:** Acaricidal plants, Cattle ticks, Indigenous knowledge, Smallholder farmers

## Abstract

**Background:**

The inclusion of traditional plant-based ecto-parasite control methods in primary health care of livestock is increasingly becoming an important intervention for improving livestock productivity in resource-challenged smallholder farming areas. In this study, commonly used plants used for the control of cattle ticks and other pests were identified through a survey in four semi–arid districts of Zimbabwe.

**Methods:**

A standard structured questionnaire with details of demographics, socioeconomic status of households, livestock parasites, control practices and list of ethnoveterinary plants used was used to interview 233 knowledgeable smallholder farmers in four districts. Focus group discussions with community members further provided insights on how the plants were being used and other issues surrounding ecto-parasite control and indigenous knowledge systems in the study areas.

**Results:**

The older generation (>40 years) of the respondents were knowledgeable about ethnoveterinary plants and practices. Overall, 51 plant species were reportedly effective against cattle ticks and other livestock parasites. The most frequently mentioned plants were in descending order, *Cissus quadrangularis* (30.1%), *Lippia javanica* (19.6%), *Psydrax livida* (14.9%) and *Aloe* sp (14.9%). Most of the plant materials were prepared by crushing and soaking in water and spraying the extract on animals. Despite the knowledge of these useful pesticidal plants, the preferred animal health care for cattle and other highly ranked livestock species is still the use of commercial acaricides. Cattle dipping services were reported sporadic by 48% of the respondents. Traditional knowledge and plants are considered only as an alternative in the absence of conventional synthetic products.

**Conclusions:**

Livestock farming communities know of plant species used for livestock ecto-parasite control. The plant species are mostly used to complement commercial products. More work, is required to confirm the acaricidal properties claimed by the farmers in order to optimize and promote sustainable use of these plants.

## Background

Livestock play a crucial livelihood role for 70% of the world’s resource-poor population whose majority are living in rural areas [1]. Their importance is even greater in drier agro-ecological zones where crop production is restricted by low and poorly distributed rainfall coupled with recurrent droughts. Mostly, these areas are only ideal for extensive livestock husbandry (especially cattle and small ruminants), wild animals and drought resistant crops. While these areas are suitable for livestock, animal performance still remains subdued due to numerous interacting factors key amongst them, diseases and poor health management across all species.

One of the major health concerns affecting livestock are ecto-parasites, particularly ticks and tick-borne diseases (TTBD). At least 80% of the world’s cattle population are at risk from TTBD [2,3]. Ticks affect cattle directly by causing skin damage opening up wounds which make the animal susceptible to secondary infection, and cause toxicosis and paralysis in some instances [4]. Indirectly and more importantly, ticks act as vectors of fatal diseases, for example babesiosis and theileriosis [5]. Minjauw and Mcleod [6] and Perry et al. [7] ranked high the effects of TTBDs on the livelihoods of resource-constrained smallholder farmers in developing countries of sub-Saharan Africa (SSA), Asia and Latin America while [8] identified TTBD as one of the most important health and management challenges in Africa ahead of tsetse fly and trypanosomiasis.

The widely accepted and most used method of ecto-parasite control has been the use of commercial chemical acaricides. In many developing countries, however, the commercial acaricides may be inconsistently available or not available at all [9]. Other challenges emanating from the use of these modern products include the development of widespread host resistance, ever-increasing cost of acaricides, environmental toxicity of chemicals, residuals in animal products and harm to non-target organisms [10]. This has compelled researchers to explore other methods that can be used as alternatives. In developing countries, there have not been significant deliberate investments of time and resources to development of tick control methods that are better suited for marginalised and resource-challenged smallholder farming communities. Most products on the market have been developed to cater for thriving commercial farmers with exotic livestock breeds which are the opposite of smallholder farmers [7]. Many of the smallholder farmers cannot easily access professional veterinary services and products and often face serious cash flow problems which make regular and sustainable purchase of commercial synthetic acaricides a big challenge. All these factors clearly indicate that there must be other ways of addressing TTBD problems other than what is currently available. Many options have been postulated including use of vaccines and integration of systems with various degrees of adoption and successes [11].

One approach is to explore and integrate the existing conventional methods of health management and ethnoveterinary practices, particularly the use of pesticidal plants. From time immemorial, people have used natural plants to control agricultural pests but the practice has been overtaken by use of synthetic pesticides and acaricides [12]. There is, however, renewed interest in pesticidal plants and traditional practices with literature suggesting that they have a great potential against agriculture pests especially for poor farmers [13]. Some of the plants with demonstrated acaricidal activity across the world include *Azadirachta indica* A. Juss. [14,15]; *Tephrosia vogelii* Hook. f. [16,17]; *Stylosanthes scarbra* Vogel, [18]; *Solanum dasyphyllum* Schumach & Thonn, [19], *Cleome gynandra* L. [20]; *Melinis minutiflora* P. Beauv. [21]. In Zimbabwe, Madzimure et al. [22,23] confirmed acaricidal properties in *Lippia javanica* leaves *(Burm.f.)* Spreng*. Solanum incanum* L*.* fruits and *Strychnos spinosa* Lam. fruits which were targeted based on an earlier ethnobotanical knowledge survey [24]. The crude water extracts of the plants reduced tick populations on cattle even though the cattle were still classified as tick-infested according to Zimbabwe veterinary standards [25].

Scientific research and documentation of medicinal and pesticidal plants is still lagging behind in most developing countries, Zimbabwe included [12]. In Zimbabwe, no surveys that cover wider parts of the country have been conducted to collect information on plants that are useful in controlling livestock parasites to broaden the knowledge and resource-base. There are potentially effective plants in different parts of the country which need to be identified. Therefore, this study was commissioned to help build a database of acaricidal plants in Zimbabwe particularly from remote semi-arid areas. This knowledge will help in further research and development of alternative pest and parasite control options for smallholders farmers in Zimbabwe and other developing countries in similar circumstances.

## Materials and methods

### Study sites

**Figure 1:**
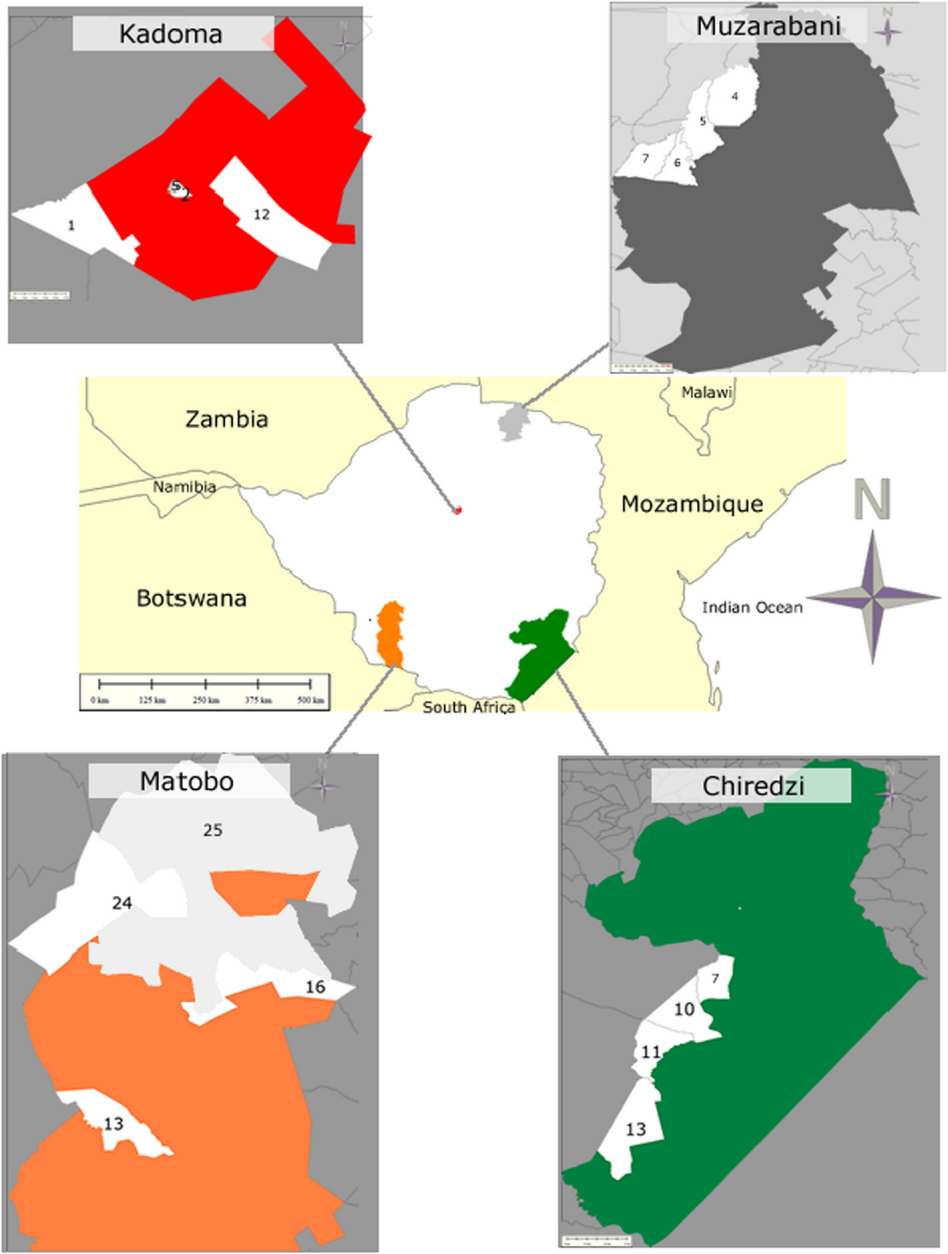
**Map of Zimbabwe showing the study sites.** The numbers withing the map denote administrative areas within the districts, locally known as Wards.

Surveys to document ethnoveterinary practices used to control ticks and other parasites in livestock were conducted in four semi-arid districts of Zimbabwe covering natural agro-ecological regions IV and V. The basis for selection of these two regions was that livestock production is key to livelihoods of farmers in these zones because of the sporadic and unreliable rainfall which make crop production and other water-dependent enterprises a huge challenge. Any intervention that helps improve livestock productivity will most likely directly improve livelihoods of these communities. In ecological region IV, Matobo and Kadoma districts were purposively selected while Chiredzi and Muzarabani districts were also purposively selected from natural ecological region V (Figure 1). In each district, four culturally-rich wards, where ethnoveterinary practices are prevalent, were targeted for administration of the questionnaire. District agricultural and veterinary extension officials assisted in identifying suitable areas to conduct the surveys. This approach was used to increase the likelihood of getting useful information since traditional practices are area-specific. With regard to human development, Matopo and Kadoma districts were ranked 36th and 43rd poorest districts in Zimbabwe, respectively; while Chiredzi and Muzarabani districts are ranked 52nd and 61st out of a total of 77 districts [26]. A short description of the study sites is presented in Table 1.

**Table 1:** **Some characteristics and description of the survey study sites**

**District**	**Ward**	**Province**	**Agro-ecological region**	**Characteristics of farming region**
Kadoma	1	Mashonaland West	III - IV	Found on approximately 18°19′S longitude and latitude 29°53′ E, moderate to low average rainfall 450 - 600 mm, semi-extensive farming of livestock and drought resistant fodder crops [27]
	2			
	5			
	12			
Matobo	13	Matabeleland South	IV	District is found at 20°23′È latitude and 28°30′S longitude. Average rainfall is 450–650 mm, area is good for semi-extensive livestock and game ranching, common crops include drought resistant maize, sorghum and millet [28]
	16			
	24			
	25			
Chiredzi	7	Masvingo	V	Area is found at about 31°30′S longitude, 21°10′E latitude. It is one of the largest districts in Zimbabwe with an average uncertain annual rainfall < 450 mm; suitable for extensive cattle production and game ranching [28]
	10			
	11			
	13			
Muzarabani	4	Mashonaland Central	V	District is located at a longitude of 16^0^E and latitude of 31^0^S along the Mozambique – Zimbabwe border. Average annual rainfall < 450 mm. The area is suitable for subsistence smallholder farming, cattle production and game ranching [28]
	5			
	6			
	7			

### Ethnobotanical data collection

A total of 233 households (60 from Muzarabani, Sanyati and Matopo districts and 53 from Chiredzi district) were purposively selected depending on whether they kept livestock or not and if they had knowledge about ethnoveterinary practices. They were interviewed using a pre-tested standard structured questionnaire. The purposive sampling technique was chosen in order to increase the likelihood of obtaining detailed and useful information from respondents. The survey was conducted between January and April 2013 and sought information on ethno tick and other livestock pest control practices and issues surrounding their use. Local agricultural and veterinary extension staff assisted in the identification, selection of respondents and subsequent administration of the questionnaires. We confirm that in Zimbabwe research surveys of the nature reported in this study did not require ethical approval by committee since they did not involve the use of the plant materials surveyed on humans or animals. All interviewees were volunteers and consented to be interviewed and approved of the subsequent publication of survey responses. Apart from the questionnaires, further information on pesticidal plants was collected through focus group discussions (FGDs) with knowledgeable persons from the respective communities. Information regarding the vernacular names, part(s) used, methods of preparation, mode of application was documented during the interviews. Each plant was correctly identified by a qualified botanist and voucher specimens deposited at the National Herbarium and Botanical Garden in Harare, Zimbabwe.

### Data analyses

Data collected were analyzed using SPSS version 21 (IBM Statistics, 2012). Frequencies, means and tables were generated for variables such as livestock species kept, major parasites affecting the livestock and the methods used for controlling them. Identified plants were ranked according to frequency of mention in all the districts. Qualitative data from FDGs were synthesised and summarised according to thematic areas.

## Results

### Household demographics

**Figure 2:**
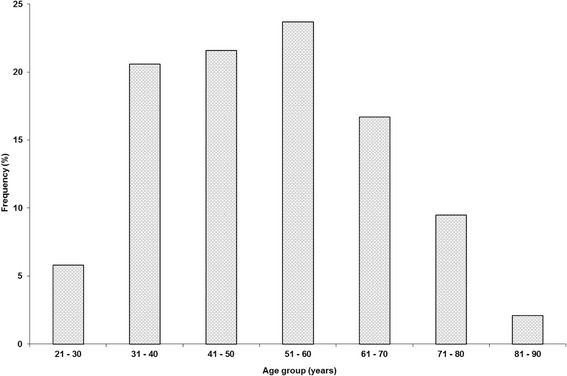
**Age group distribution of respondents across the surveyed districts (** ***N*** **= 233).**

A total of 233 household heads responded to the questionnaire in the four districts of which 78.5% were males and 21.5% were females. Their average age was about 51 years and the household size averaged 7 people (Table 2). The age distribution of respondents showed that they were mostly of the older generation with the bulk of them over 40 years of age (Figure 2). About 6% were found in the youngest age group (21 – 30) and only 2% were in the oldest group (81 – 90).

**Table 2:** **Summary of household demographics (N = 233)**

**Household details**	**Mean**	**Std. Deviation**
Age of household head (years)	51.3	15.23
Household size	7.2	4.46
Number of adult males (>18 years)	2.0	1.52
Number of adult females (>18 years)	2.0	1.57
Number of children (0–18 years)	3.4	2.62

### Livestock species kept by the farmers

**Figure 3:**
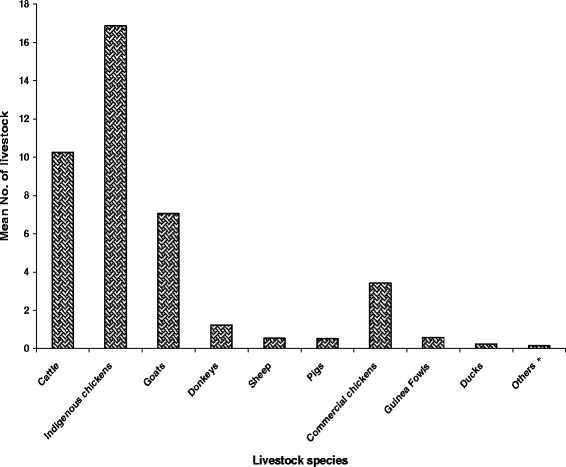
**Average livestock numbers kept per household across the survey districts (** ***N*** **= 233).** *Others represents small livestock species like pigeons, rabbits and rock rabbits kept in small numbers.

**Figure 4:**
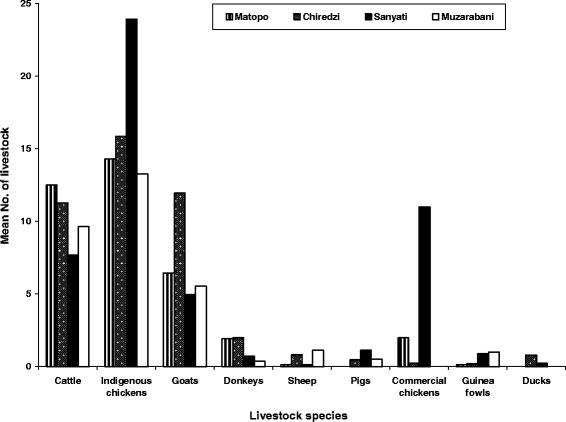
**Mean number of livestock species by district (N = 233).**

Most households owned different types of livestock which consisted mainly of poultry, cattle, goats, sheep, donkeys and pigs. Indigenous chickens were the most populous species kept per household with a mean estimate of 17 chickens (Figure 3). The highest population of cattle was found in Matopo district while goats were most populous in Chiredzi. There were more indigenous chickens kept in Kadoma than any other district. Across the districts, the mean number of sheep was very low. A detailed picture of the distribution of the livestock species by district is as shown in Figure 4.

### Ranking of animal species

Despite chickens being the most populous species kept by respondents, cattle were ranked highest of all the species in terms of importance followed by indigenous chickens and then goats (Table 3). Other species kept by respondents in the survey areas and ranked lower include commercial chicken breeds, pigs, donkeys and various other less popular poultry species like guinea fowl and ducks.

**Table 3:** **Ranking of livestock species according to importance by respondents (N=233)**

**Species**	**Rank**	**Frequency (%)**
Cattle	1	77.7
Indigenous chickens	2	76.9
Goats	3	56.6
Donkeys	4	28.4
Sheep	5	13.8
Pigs	6	10.7
*Other species	7	7.2
Commercial chickens	8	5.7

*Refers to livestock species that were available in very small numbers which include; turkeys, ducks, rabbits, pigeons and guinea fowls.

### Prevalent livestock parasites and their management

**Figure 5:**
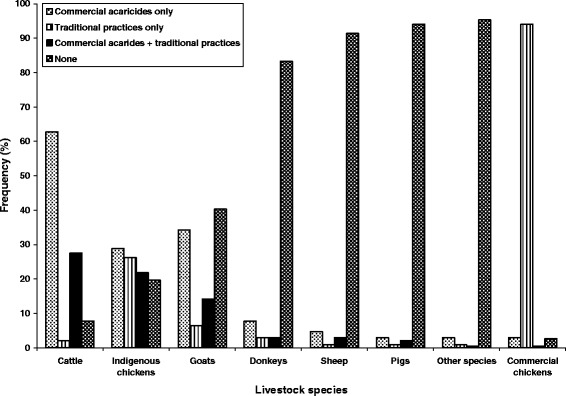
**Livestock ecto-parasites management techniques by livestock species in the surveyed districts (N = 233).**

Ticks were the most commonly identified parasites affecting mostly cattle, goats, donkeys and sheep (Table 4). Other parasites also reported include common flies and tsetse flies. Mites, fleas and lice were the most prevalent parasites in poultry species with the most common parasites for indigenous chickens being fleas and lice at 27.5% and 22.7% respectively (Table 4). Generally, parasites were controlled mostly by synthetic acaricides only for the highly ranked animal species (cattle, indigenous chickens and goats) with the lowly ranked animals receiving little or no remedial action at all (Figure 5). The use of traditional practices alone was prevalent mainly in poultry production compared to other systems. A mixture of traditional practices and commercial products was prevalent mostly in cattle, indigenous chickens and goats. It was, however, minimal in pigs, sheep and donkeys (Figure 5).

**Table 4:** **The most frequently mentioned livestock parasites by species in the four districts**

**Livestock Species**	**Common parasites as identified by respondents (%)**					
	**Ticks**	**Flies**	**Tsetse flies**	**Mange mites**	**Fleas**	**Lice**
Cattle	81.0	0.9	0.4	0.4	-	-
Sheep	5.6	0.4	-	-	-	-
Goats	49.8	-	0.4	3.9	0.4	0.4
Indigenous chickens	-	1.3	-	3.4	27.5	22.7
Commercial chickens	-	-	-	-	0.4	0.9
Pigs	-	-	-	2.2	-	1.3
Donkeys	13.3	0.4	-	-	0.4	0.4
*Other species	-	0.9	-	-	0.9	2.1

*Refers to livestock species that were available in very small numbers. These include; turkeys, rabbits and guinea fowls. "-" means the ectoparasites were reported absent for that livestock species.

### Status of cattle dipping in the survey areas

**Figure 6:**
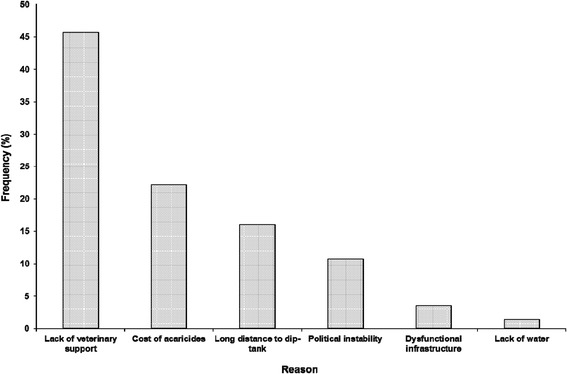
**Reasons cited for inconsistent dipping services in the survey areas (N = 233).**

A significant percentage of the respondents highlighted that dipping services were sporadic (48%) and in some areas not available at all (12%). However, 40% of the respondents had no challenges with availability of dipping services. Chief amongst the reasons cited for inconsistent dipping services was the lack of veterinary services through Government support and high cost of acaricides (Figure 6). Other reasons included long distances to dip tanks, political instability, dysfunctional dipping infrastructure and unavailability of water.

### Use of pesticidal plants

**Figure 7:**
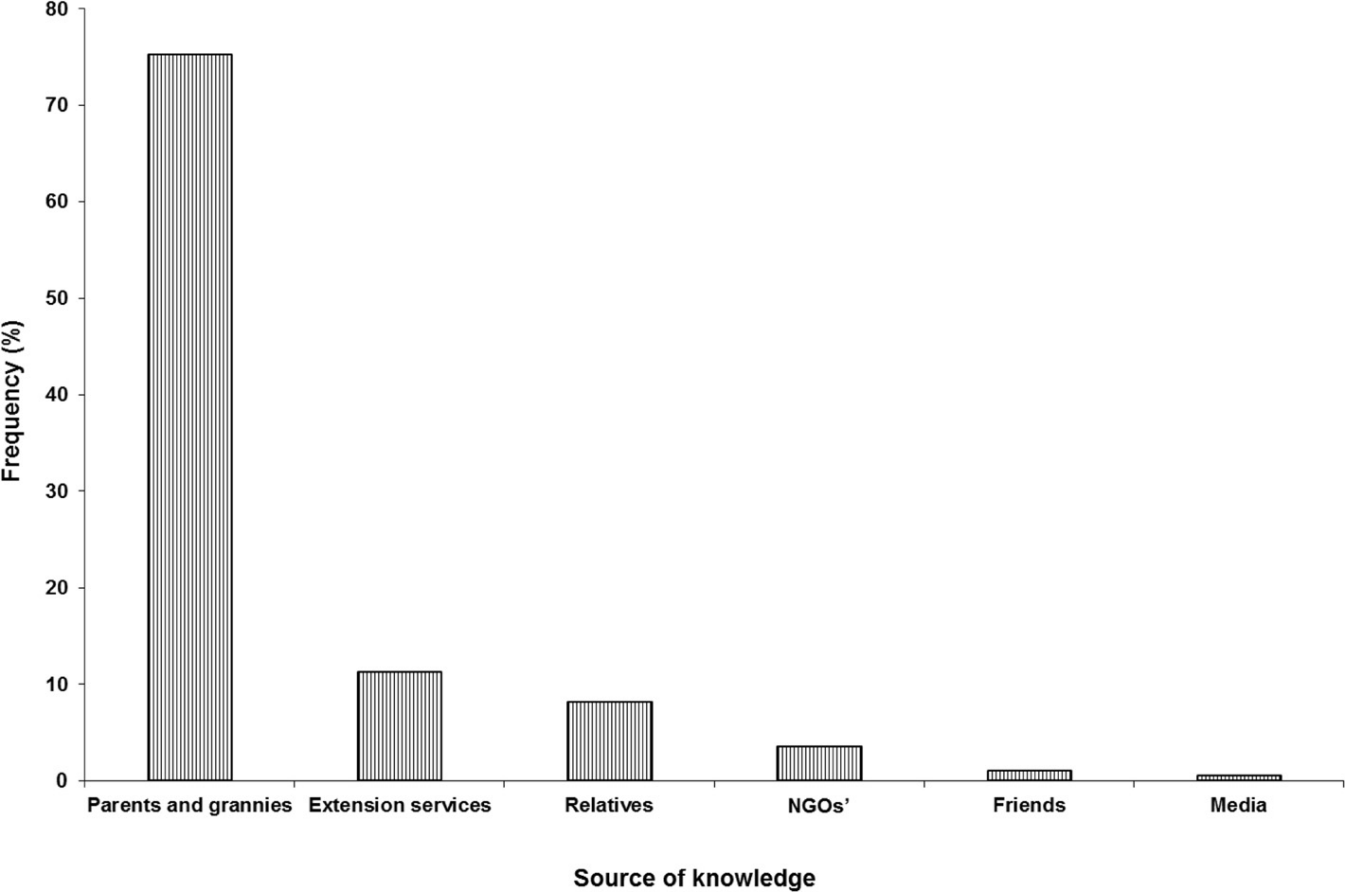
**Sources of knowledge of acaricidal plants in the survey areas.**

Many respondents (72.1%) had some knowledge of plants used in the control of animal parasites whereas 27.9% were totally unaware. The knowledge was passed on orally mainly from the older generation of parents and grandparents (75.3%). Other knowledge sources of pesticidal plants were local agricultural extension staff, relatives, Non-Governmental Organisations (NGOs), friends and the media in descending order (Figure 7).

**Figure 8:**
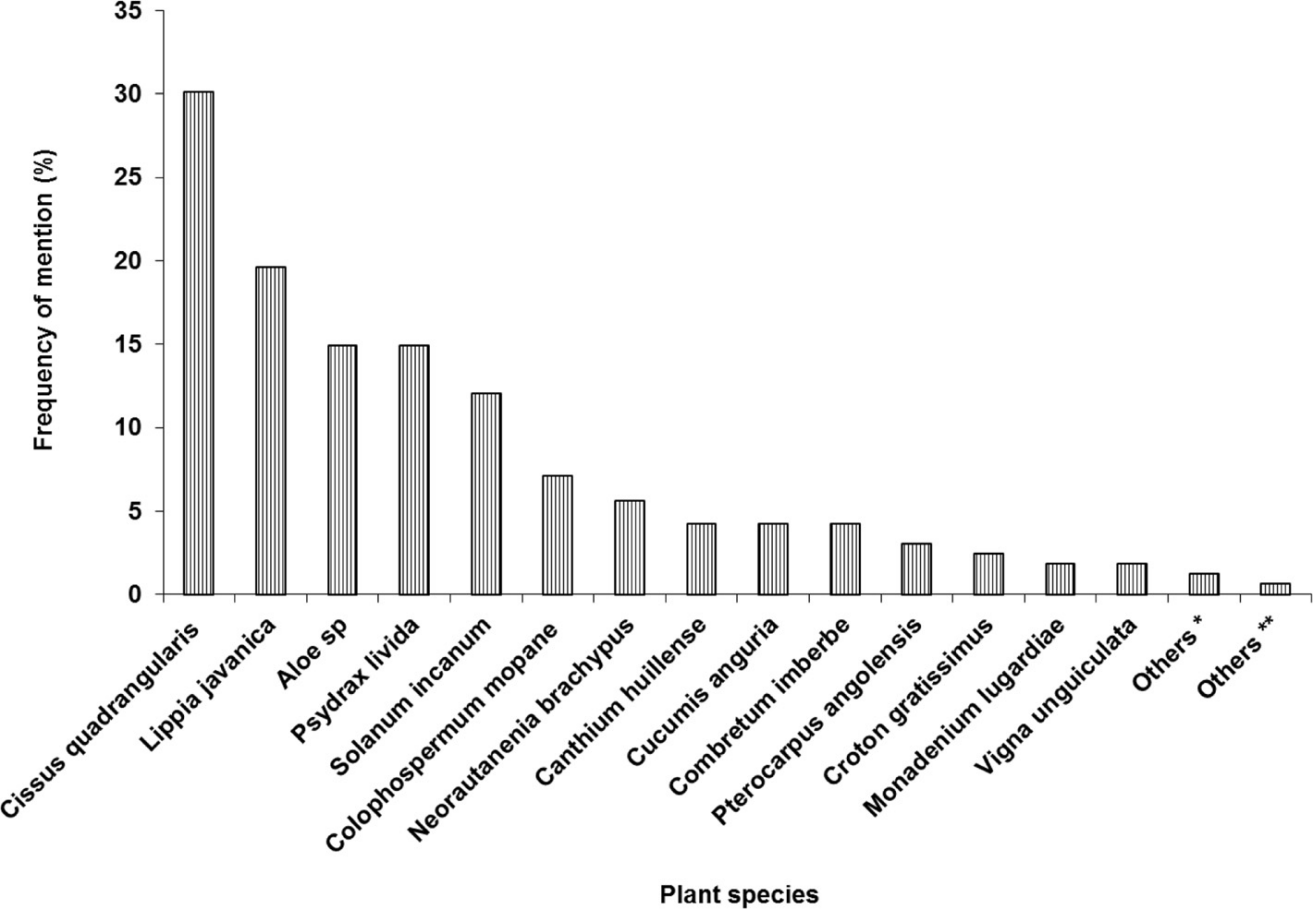
**The most common acaricidal plants by frequency of mention (N = 233).** * Plant species with a frequency of 2: *Acacia karoo, Bauhinia petersian, Capsicum annuum, Clerodendrum eriophyllum, Vernonia colorata, Spirostachys africana, Strychnos spinosa, Terminalia sericea, Strychnos cocculoides, Zantedeschia albomaculata*. ** Plant species with a frequency of 1: *Albizia harveyi, Carissa edulis, Ornithogalum sp, Chimwamaruka, Euphorbia griseola, Gnidia kraussina, Kleinia sp, Jatropha curcas, Maerua edulis, Euclea divinorum, Mundorani, Rotheca eriphylla, Nicotiana tobaccum, Olinia ventosa, Sansevieria hyacinthoides, Senna singuena, Tagetes minuta, Xeroderris stuhlmannii, Zanthoxylum chalybeum*.

Overall, a total of 51 different plant species effective against livestock parasites were identified from the survey. The most popular plants by frequency of mention were *Cissus quadrangularis* L. (30.1%), *Lippia javanica* (19.6%)*, Psydrax livida* Willd. (14.9%) and *Aloe* sp (14.9%) (Figure 8). *Cissus quadrangularis* was particularly singled out as a very effective plant acaricide in Muzarabani, Kadoma and Chiredzi districts in FGDs with the farmers. Many other plant materials were not as well-known and were less mentioned by the respondents (Figure 8). The information on the specific pesticidal plants (trees, shrubs and grasses), preparation methods, parts of the plant used, targeted parasites, availability and other issues based on farmers’ experiences are summarised in Table 5. The most common method of preparation of the plant materials was crushing the leaves/stems, soaking in water for variable periods and then spraying the animals. Other methods included dusting ashes of certain trees, shrubs and herbs over the animals and birds. In poultry, twigs and leaves of *L. javanica* were laid as bedding in the fowl run.

**Table 5:** **Summary of plants used for ticks and other ecto parasites control, how they are used and status of availability**

**Scientific name & Voucher number**	**Family name**	**Local names** ***Shona-S Ndebele-N Shangani-C***	**Part used**	**Preparation methods**	**Target parasites**	**Availability status**	**Comments & precautions**
*Acacia karroo* Hayne.	Fabaceae	Muzunga (S)	Root	Leave root in fowl run	Fleas and mites	Always	Safe to use
*Nyahangare E 11*							
*Monadenium lugardiae* N.E. B.	Euphorbiaceae	Chisvosve (S)	Whole plant	Crush and mix with water 24 h	Ticks, fleas	Always	Handle with care
*Nyahangare E 15*							
*Albizia amara (*Roxb.)Boiv.	Fabaceae	Umbola (N)	Leaves	Crush leaves + water + spray	Ticks, fleas	Seasonal	Effective
*Nyahangare E 38*							
*Aloe excelsa* A. Berger.	Aloaceae	Gavakava, Mhangani(S)	Stemmy leaves	Crush leaves, mix with water for 24 h & spray	Fleas, ticks	Seasonal	Safe to use
*Nyahangare E 29*							
*Aloe chabaudii* Schoenland.	Aloaceae	Inhlaba (N)	Succulent leafy stems	Grind and soak and smear the bird with sticky water	Lice	Always	Very effective
*Nyahangare E 37*							
*Bauhinia petersiana* Bolle.	Fabaceae	Mutyatyambe (S)	Leaves	Crush leaves, mix with water	Ticks, Goats	Always	Safe to use
*Nyahangare E23*							
*Capsicum annuum L.*	Solanaceae	Mhiripiri (S)	Fruits	Crush the fruits and mix with soot in water and spray	Ticks	Always	Causes eye irritation
*Nyahangare E 69*							
*Carissa edulis (Forssk.)* Vahl*. Nyahangare E39*	Apocynaceae	Umlugulu (N)	Leaves	Grind leaves, mix with water in the ratio 1:4 and spray	Lice and ticks	Seasonal	Wash hands after use
*Cucumis anguria L.*	Cucurbitaceae	Mujachacha (S) Amagaka (N)	Fruits	Collect ripe fruits (yellow), crush and mix with water and spray	Ticks	Seasonal	Have an itching effect on animals
*Nyahangare E47*							
*Ornithogalum sp*	Asparagaceae	Chihanyanisi (S)	Roots	Crush and mix with water	Ticks	Always but scarce	Very effective
*Nyahangare E59*							
*		Chimwamaruka (S)	Leaves and branches	Crush the leaves and branches and mix with water	Fleas and ticks	Always but scarce	Very effective
*Cissus quadrangularis L.*	Vitaceae	Chiololo (C), Murunjurunju (S)	Stems	Crush and mix with water to spray	Ticks	Always	Handle with care, has an itchy effect
*Nyahangare E6*							
*Psydrax livida (*Hiern) Bridson	Rubiaceae	Muvengahonye (S) Umhlahlampethu (N)	Leaves	Crush, mix with water and spray or crush leaves and put on wound or tick infestation site	Ecto parasites	Always	
*Nyahangare E20*							
*Rotheca eriophylla*	Lamiaceae	Umnukanja (N) Munukanja (S)	Leaves	Macerate, soak with water and spray	Ticks, Lice and tsetse fly	Always	Very effective and safe to use
*Nyahangare E35*							
*Colophospermum mopane* (J. Kirk ex Benth.) J. Kirk ex J. Leonard.	Fabaceae	Mopani (S)	Branches and twigs	Burn and apply ashes on animal skin	Ticks, fleas, mites	Always	Safe to use
*Nyahangare E41*							
*Combretum imberbe* Wawra.	Combretaceae	Muchenarota/Mutsviri (S)	Bark	Ash of the bark and twigs dusted on infestation sites	Ticks	Always	Very effective
*Nyahangare E55*							
*Croton gratissimus Burch.*	Euphorbiaceae	Inkiza emhlope (N)	Leaves and twigs	Use as bedding in the fowl run	Lice	Always	Very effective
*Nyahangare E48*							
**Scientific name & Voucher number**	**Family name**	**Local names** ***Shona-S Ndebele-N Shangani-C***	**Part used**	**Preparation method**	**Target parasites**	**Availability status**	**Comments & precautions**
*Ptaeroxylon obliquum (*Thunb.) Radlk.	Rutaceae	Umpahla, Umpandula N,	Leaves	Crush leaves, soak in water and spray	Ticks, Fleas, Lice	Always but scarce	Very effective
*Nyahangare E27*							
*Euphorbia cooperi L.*	Euphorbiaceae	Umhlonhlo (N)	Branches	Smoke the walls of the fowl run with the branches	Fleas	Always	Handle with care
*Nyahangare E71*							
*Euphorbia ingens* E.Mey.ex Boiss.	Euphorbiacaea	Mukondekonde (S)	Stems	Mix milk sap with water and place on infested sites	Fleas, ticks	Always	Avoid contact with the eyes
*Nyahangare E43*							
*Gnidia kraussiana* Meisn.	Thymelaeaceae	Chitupatupa (S)	Whole plant	Crush and mix with water to spray	Ticks	Always	Very effective
*Nyahangare E70*							
*Lippia javanica* (Burm.f.) Spreng.	Verbenaceae	Zumbani (S) Umsuzwane (N)	Leaves and branches	Crush leaves mix with water and spray, twigs can also be used as bedding in fowl runs	Ticks, fleas, lice	Always	Safe to use
*Nyahangare E46*							
*Maerua edulis* Gilg&Gilg-Ben. DeWolf	Capparidaceae	Katunguru (S)	Roots	mix *M. edulis* roots and *S. incanum* fruits with water and spray	Ticks	Always	Very effective
*Nyahangare E5*							
*Ornithogalum sp*	Alliaceae	Masimbe (S)	Bulb	crush bulb, soak in water and spray	Ticks	Always	Very effective
*Nyahangare E59*							
*Datura stramonium* L.	Solanaceae	Iyoyi (N), Chowa (S)	Leaves	Crush leaves mix with water and spray on animal	Ticks and Lice	Always but scarce	Safe to use
*Nyahangare E51*							
*Euclea divinorum* Hiern.	Ebenaceae	Muchekesani(S)	Roots	Crush leaves soak in water and spray	Fleas, cattle	Always	
*Nyahangare E15*							
*		Mundorani (S)	Roots	Crush dry roots and mix with water (50 g in 300 ml for 3 days)	Ticks	Always but scarce	Very effective
*Vernonia colorata* (Willd.) Drake.	Compositae/ Asteraceae	Munyatera (S)	Roots	Crush mix with water for an hour and spray	Ticks	Always but scarce	Very effective
*Nyahangare E16*							
*		Muvuwavuwa (S)	Whole plant	Just put the plant in the fowl run	Fleas and lice	Always but scarce	Very effective
*Neorautanenia brachypus*	Fabaceae	Zhombwe (S)	Bulb	Crush the bulb, mix with water and spray	Fleas, ticks	Always	Avoid water contamination
(Harms) C.A. Sm.							
*Nyahangare E65*							
*Nicotiana tabacum* L.	Solanaceae	Tobacco (E)	Water	Break and mix with water	Ticks	Always	Safe to use
*Nyahangare E68*							
*Spirostachys africana* Sond.	Euphorbiaceae	Mutovhoti (S)	Barks	Cut barks break and mix with water	Ticks	Seasonal	Very effective
*Nyahangare E63*							
*Strychnos spinosa* Lam.	Strychnaceae	Matamba (S)	Unripe fruits	Crush unripe fruits, mix with water and spray	Ticks	Always	Very effective
**Scientific name & Voucher number**	**Family name**	**Local names** ***Shona-S Ndebele-N Shangani-C***	**Part used**	**Preparation method**	**Target pests**	**Availability status**	**Comments & precautions**
*Nyahangare E10*							
*Tagetes minuta* L.	Compositae/ Asteraceae	Munyakambanje (S)	Whole plant	Crush and mix with water	Ticks, fleas, mites	Always	Safe to use
*Nyahangare E66*							
*Terminalia sericea* Burch. ex DC.	Combretaceae	Mususu (S)	Leaves	Crush mix with water for spray	Ticks	Always	Very effective
*Nyahangare E36*							
*Strychnos cocculoides* Baker.	Strychnaceae	Ubuhlali (N)	Fruit	Squash the fruit and smear contents on animal infested	Lice	Seasonal	Very effective
*Nyahangare E45*							
*Vigna unguiculata* L.	Leguminosae - Papilionoideae	Cowpea (E)	Pods	Empty pods burnt and ash applied on tick sites	Ticks	Always	Very effective
*Nyahangare E67*							
*Xeroderris stuhlmannii*	Papilionaceae	Murumanyama (S)	Barks	Crush stems and spread on infestation sites	Fleas, ticks	Always	Very effective
*(*Taub.)Mendonca &E.P.Sousa *Nyahangare E7*							
*Zantedeschia albomaculata* (Hook.) Baill.	Araceae	Mufanawembudzi (S)	Stem	Crush, mix with water and drench	Ticks	Always	Very effective
*Nyahangare E62*							
Zanthoxylum capense (Thunb.)Harv. Nyahangare E22	Rutaceae	Mukundanyoka (S)	Stems	Crush stems and spread on infestation sites	Fleas, ticks	Always	Safe to use
*Xanthocercis zambesiaca* (Baker) Dumaz-le-Grand *Nyahangare E8*	Fabaceae	Muturufuwa (S)	Bark	Crush the bark and soak in water to form a soapy solution for spraying	Fleas and ticks	Available	Safe to use
*Pterocarpus angolensis DC. Nyahangare E32*	Papilionoideae	Umvagazi (N) Mubvamaropa	Barks, branches	Mix with water	Ticks	Always	Safe to use
*Sansevieria hyacinthoides (L.) Druce. Nyahangare E58*	Asparagaceae	Chikwenga (S)/Bushfibre (E) Mashamhanda (S)	Stemmy rhizomes	Squash the stems, mix with water and spray on ticks	Ticks	Always	Handle with care
*Senna singueana (Del.) Lock Nyahangare E13*	Fabaceae	Mudyamhungu Mukundanyoka (S)	Bark	Crush bark and mix with water	Ticks	Always	User friendly
*Solanum incanum L. Nyahangare E61*	Solanaceae	Nhundurwa (S)	Fruits	Crush fruits and mix with water	Ticks	Seasonal	Handle with care
*Solanum panduriforme E.Mey. Nyahangare E60*	Solanaceae	Nhundurwa (S)	Fruits	Crush fruits and mix with water	Ticks	Seasonal	Handle with care
*		Chinyaride (S) (fibre like)	Roots and the bulb	Crush the roots/bulb and mix with water	Ticks	Always but scarce	Handle with care
*Kleinia sp Nyahangare E50*	Asteraceae	Iphunja (N)	Leaves	Crush leaves, mix with water and rub on affected areas	Ticks	Scarce	Very effective
*Ricinus communis L. Nyahangare E42*	Euphorbiaceae	Umtshafuto (N)	Leaves	Grind leaves and paste on tick infested site	Ticks	Always	Very effective
*Osyris lanceolata Hochst. Nyahangare E49*	Santalaceae	Ingobamakhosi (N)	Roots	Pestle roots, soak in water and spray	Ticks	Always but scarce	Very effective

* *At the time of the survey, samples of these plants could not be found and require further follow up for positive identification.*

### Focus group discussions

During discussions, farmers acknowledged that they were more knowledgeable about livestock medicinal plants than pesticidal plants in general. The practice of using ashes of different plants as acaricides, for example *C. mopane* tree, was based on the observation that donkeys regularly roll or bath in ashes in the villages and consequently, are rarely tick-infested despite not being dipped at all. Farmers in Muzarabani district showed awareness of aspects to do with intellectual property rights from previous exposure to studies on medicinal plants by other researchers. They felt that the scientists took advantage of them by extracting information from them and making money out of it with no acknowledgement of their input in monetary terms or otherwise. They demanded assurance that whatever came out from the deliberations would remain community property and that they should benefit as well. Separately, there was concern by farmers in all the districts about political interference and general lack of government support to arrest deteriorating health care provision to animals. In newly resettled areas, farmers lamented the long distances they must travel to access communal dip tanks.

## Discussion

### Household demographics

The study showed that most of the respondents were of an older age ranging between 41–70 years of age. Similarly high, was the mean age (51.3 years) of the household head. This is due to the purposive nature of the sampling where the focus was on livestock farmers and younger generations were unlikely to own livestock as they were in the process of pursuing an educational career or other developmental avenues of life. The older generation were also more knowledgeable about the use of traditional plants in the provision of primary health care of animals. It is known that traditional knowledge and practices are normally found in the older generations as the younger generations generally disregard traditional practices as a result of direct and indirect effects of modernisation and globalisation [29-31].

### Livestock production in the survey areas

Overall, indigenous chickens were the most populous livestock species averaging 16.9 birds per household which compares well with the national average in rural areas of Zimbabwe of around 17 birds [32]. Mutibvu and co-workers [33] also found an average of 16.1 birds per household in a survey carried out in Simbe Communal area of Gokwe South district of Zimbabwe. Indigenous chickens generally require minimal initial capital injection compared to other species and can also perform relatively well even under poor management. Almost every farmer can afford to raise the birds on the farm regardless of social status. Chickens provide the household with readily available cheap protein and can also be used as a form of quick off-take, thus playing a critical role in the livelihoods of farmers [34]. Kadoma district had the highest mean average of chickens kept per household. This can be explained by the fact that indigenous chickens also rely, to a lesser extent, on maize, sunflower and other grains. In Kadoma district, crop production is most favourable compared to the other districts.

Despite chickens being the most populous species, cattle were ranked as the most important livestock species on the farms. Cattle are considered very important because of the many socio-cultural and economic roles they play in the African society. They have multiple uses for smallholder farmers providing draught power; used in traditional rituals, providing milk, meat, manure and acting as social security among other important cultural roles [35-38]. The importance of cattle is also underscored by other researchers and [39,40] who made similar observations. The other livestock species were ranked lower and had lower mean herd and flock sizes. Muchadeyi [32] reported that even though farmers keep other species such as commercial chicken breeds, pigs, donkeys and various other less popular poultry species like guinea fowl and ducks, they are generally ranked lower and their populations are also very low. Despite being lowly ranked, these species play important roles in the livelihoods of rural farmers. Donkeys for example were ranked fourth but are critical in the drier areas of the country where they provide a steady supply of draught power [41]. They are well-known to be drought tolerant and easier to manage than cattle. The study areas experience frequent droughts hence donkeys occupy a very important niche. The only disadvantage of donkeys is that while other domesticated animal species can be used for milk and/or meat, not many people are known to prefer donkey products in Zimbabwe.

### Livestock parasites and control methods

#### Common livestock parasites and pests identified in the survey

Most farmers identified ticks as the most problematic pest in cattle and other animals. Other authors [42,43] also found ticks to be the major problematic external parasite in cattle-rearing in South Africa and Zimbabwe respectively. There is a long history of the negative effects of ticks on productivity of cattle. Young [8] put the economic effects of ticks in SSA higher than tsetse flies. The effects of other parasites are not as high and debilitating as those of ticks which can cause several weakening and fatal conditions to the animals [44,45]. The high incidence of fleas and lice in poultry is not uncommon as these are the most common parasites affecting chickens. Maroyi [43] also reported a high presence of these pests in chickens in a survey in Nhema area of the Midlands province of Zimbabwe.

#### Methods of controlling parasites

There was a deliberate inclination to manage ecto-parasites in the highly ranked livestock species (cattle, indigenous chickens and goats) with commercial acaricides while there were few interventions in the other livestock species with some of them not receiving any attention at all. The preference of commercial acaricides can be explained by the fact that over a long period, commercial products have been demonstrated to be very effective and are thus the default pest control remedy. On the contrary, farmers perceive the efficacy of traditional practices to be on the low side even if those thoughts are not supported by hard scientific facts [46]. Government of Zimbabwe has also played a critical role in the promotion of commercial products over traditional practices through extension agencies as Government policy. In most developing countries, there is no formal policy on the use of pesticidal plants and traditional practices in general. It must also be noted that through the Department of Veterinary Services of Zimbabwe most acaricides are heavily subsidised for communal farmers [47]. This implies that, for as long as the services are available farmers will take this route for animal health care. It is when commercial acaricides are not available that farmers start using plants and other ethnoveterinary practices. Isman [13] noted that use of pesticidal plants is most prominent in the absence of commercial pesticides due to various reasons.

Challenges arise when governments no longer have capacity to meet this need and instances of this nature are occurring more frequently. Between the years 2000 and 2009 for example, Zimbabwe went through serious economic challenges and cattle herds would go for months without dipping because the government could not supply the required chemicals [48]. Prior to the economic challenges, community dipping infrastructure was also dilapidating due to neglect of the past three decades [49]. Availability of acaricides has improved slightly as a result of economic stability over the last couple of years. Failure of governments to meet the needs of farmers is not a Zimbabwean problem alone but a feature in other countries as well; for example in some South African communities [50].

The pronounced use of acaricidal plants in combination with commercial acaricides observed in the survey shows the complementarity of the two practices in holistically dealing with ectoparasites. Other researchers have reported this trend in several ethnoveterinary studies [42,46,51].

It is not surprising that the major reason for inconsistent dipping was associated with lack of Government support. As alluded to before, farmers had become used to Government providing the acaricides and ensuring that animal health is well-catered for through the Department of Veterinary Services but this has since changed as the economic climate is changing and Government ministries are always under-funded. As farmers try and adapt to the new set-up, it is inevitable that they feel the acaricides are over-priced and that the Government is not fully supporting them. The issue of inadequate water supply as another challenge is likely to increase as more frequent droughts and erratic rainfall patterns are anticipated due to the effects of climate change. The design of the plunge dip tanks in the country is such that they must be filled with water (about 20 000 litres) so that many cattle can be immersed when they dip in. In the absence of sufficient quantities of water, it will be impossible to dip the cattle.

#### Use of acaricidal plants for control of parasites

The high proportion of respondents (72.1%) with knowledge of plants with pesticidal properties is perhaps indicative of a very good link between primary animal health care and traditional practices. A 100% affirmation to knowledge of pesticidal plants was anticipated but it became apparent during the survey that some respondents had confused pesticidal plants with medicinal plants. Nonetheless, the knowledge can be explained by that many people (>80%) in the world rely on traditional practices to address human health problems [52]. Plants are a critical component of traditional medicines and pesticides [53]. Most of traditional knowledge is not as yet comprehensively documented and is passed on orally from generation to generation which could be the reason why parents and grandparents are the biggest source of this knowledge in this study. In a study on ethnoveterinary medicines [51], 61.5% of the sampled population also reported getting knowledge from parents and grandparents. The various media platforms were cited as the least significant source of information but it must be noted that it can be the most powerful tool in the promotion and development of traditionally based remedies especially if the younger generation are to be involved.

Some of the plants that were reported were well-known while others were known by a few people. In Table 5, there is a possibility of inter-use of the plants identified. Some are stated as useful in controlling fleas, lice, flies etc. with no specific mention of ticks but it is possible that the pesticidal properties can also work against ticks. Future studies should investigate this possibility. The most common plants across the districts were *C. quadrangularis*, *L. javanica*, *P. livida* and *Aloe* sp. It is important to note that it does not necessarily mean that the most mentioned plants are the most effective as only efficacy experiments can determine that. Some plant species have over time, been well-known to work in different places of the world thus exposing them to many people. The most popular plants can help researchers prioritise plants for further investigations to meet farmer needs. Knowledge of traditional products is a trade which some people depend on for livelihood and it is very likely that such people are reluctant to divulge and share the information. This could explain why some plants could be less popular. Some of the species in the list have since been confirmed through experimentation to be acaricidal for example, *L. javanica* [22], *S. spinosa* and *S. incanum* [23]. *Aloe ferox* was reported as a tick control remedy in some South African villages [42]. *Aloe chabaudii, N. mitis* are also some examples of plants reported in another survey in Nhema village in Zimbabwe to be acaricidal [43].

Other plant species reported in this survey, however, do not have known acaricidal or pesticidal properties yet but have other uses in ethnoveterinary medicine. *Aloe* sp are effective anti-microbials and mostly used in the treatment of poultry diseases and other internal parasites [51,54,55]. The recorded uses of *C. quadrangularis* are of a medicinal nature than pesticidal [56]. However respondents highly regarded this plant and in Chiredzi it was called “*Chiololo”* in Shangani which can be literally translated to “highly effective” in English. *Psydrax livida* belongs to a very important group of plants locally known in Shona as “*Muvengahonye*” (hater of maggots) with many uses in ethnoveterinary medicine in tropical Africa but mainly famed for their antiseptic properties and treatment of wounds [43,57,58]. Other plants in the group include *Canthium huillense* and *Clausena anisata.* There have not been reports of these plants against ticks elsewhere but some farmers indicated that the leaves are excellent insect and tick repellents.

### Other issues

The use of water and leaves is almost a standard practice in most traditional remedies. Leaves are ideal because they ensure sustainability of the plant and water is a cheap universal solvent. In further studies, there may be need to use organic solvents to fully optimise the extraction process because water has its polarity limitations [59,60]. In all the districts, through the focus group discussions, most farmers were not comfortable sharing their ethno-knowledge for fear of exploitation but later relaxed their stance after much persuasion and assurance that the research was not for commercial gain. Trust is therefore a critical factor in accessing detailed and accurate information. This just shows some of the challenges that need to be addressed to fully develop ethnoveterinary science in pest control.

## Conclusions and recommendations

The study showed that there is still a considerably strong use and wide knowledge of plant-based materials in controlling cattle ticks and other livestock parasites in drier parts of the country. A total of 51 plant species were identified that can be used to control ticks in semi-arid areas of Zimbabwe but the most popular across the four districts were *C. quadrangularis, Aloe* sp and *Psydrax livida*. The foliage of these plants is reportedly crushed and mixed with water after which the extracts are sprayed on animals. There is need, however, to go a step further and conduct safety, efficacy and optimisation trials to verify farmers’ claims that the plants are safe and effective for promotion of adoption. It is also of paramount importance to start engaging the Government of Zimbabwe to explore ways of creating an enabling environment for the formal recognition, development and use of acaricidal plants and other ethnoveterinary practices in the primary health care of livestock. This should be a critical step in the development of a robust alternative or integrated system for managing cattle ticks, especially where veterinary services are limited. Intellectual property rights are an area of concern that needs to be investigated and addressed. Since the current type of work is still not yet fully developed locally, there may be need to examine what models other countries strong in the use of indigenous knowledge systems are using and assess if such models can also be adapted locally.
